# The question of strains in AA amyloidosis

**DOI:** 10.1038/s41598-025-87239-6

**Published:** 2025-01-29

**Authors:** Gunilla T. Westermark, Ebba Nyström, Sofie Nyström, K. Peter R. Nilsson, Per Hammarström, Per Westermark

**Affiliations:** 1 Department of Medical Cell Biology, Uppsala University, 75123 Uppsala, Sweden; 2 Department of Physics, Chemistry and Biology (IFM), Linköping University, 58183 Linköping, Sweden; 3 Department of Immunology, Genetics and Pathology, Uppsala University, Rudbeck Laboratory, C11, 75185 Uppsala, Sweden

**Keywords:** Fluorescent amyloid ligands, Amyloid transmission, Prion, Protein aggregation, Amyloid strains, Biochemistry, Biological techniques, Molecular biology, Diseases, Molecular medicine, Pathogenesis

## Abstract

The existence of transmissible amyloid fibril strains has long intrigued the scientific community. The strain theory originates from prion disorders, but here, we provide evidence of strains in systemic amyloidosis. Human AA amyloidosis manifests as two distinct clinical phenotypes called common AA and vascular AA. Glomerular amyloid deposition of the kidney defines the common form, while in the vascular type amyloid deposits are massive in the renal medulla and in arteries throughout the body, while glomeruli are spared. By electron microscopy the two types appeared morphologically different. The common type was composed of dispersed fibrils which tended to be clustered whereas the vascular type was composed of longer and more distinct less clustered fibrils. Staining with fluorescent amyloid binding ligands analyzed by hyperspectral microscopy showed differential staining patterns between the two groups supporting the notion of human AA amyloid strains. AA amyloid staining was significantly different from systemic AL amyloid. Both types of AA (common and vascular) and AL amyloid fibrils were isolated and used to seed mouse AA amyloid in groups of inflamed NMRI mice (*n* = 9–10 per group). All but two mice showed amyloid deposits in the spleen induced by the human seeds. Amyloid binding ligand analysis was applied on the splenic amyloid deposits and revealed no clear significant difference between mice seeded with AA fibrils from different donors being vascular or common, but the AA deposits of mice given AL fibrils showed significantly different amyloid fluorescent signals compared to all groups of mice receiving AA fibrils. The combined results support the hypothesis that AA amyloid fibril structures can vary depending on the seed and may manifest as amyloid strains.

**Supplementary Information:**

The online version contains supplementary material available at 10.1038/s41598-025-87239-6.

## Introduction

Amyloid fibril propagation is similar to that of prion spreading. Prions are misfolded infectious proteins that in human cause lethal neurodegenerative diseases, including Creutzfeldt-Jakob disease and Kuru and in animals spongiform encephalopathy^1^. Although it is the same protein that adopts infectious properties, there are several distinct prion diseases. The reason for this seems to be the ability of the prion protein (PrP) to adopt differences in folding, referred to as ‘strains’^2^. Strains propagate by a templating mechanism. Variability in misfolding of amyloid proteins is not restricted to prion proteins. It was found that Aβ protein can aggregate into several fibrillar forms that may be serially propagated in vitro^3,4^. Importantly, extracts of Alzheimer disease brains seed Aβ fibrils in vitro^5^ and fibrillar Aβ material from mutant and sporadic Alzheimer brains induce aggregates in transgenic mouse brains that differ, as shown with conformation-sensitive fluorescent ligands^6^. Also, α-synuclein seems to aggregate into distinct strains and these are correlated to different α-synucleinopathies^7^. In addition, there may be several tau strains^8^.

Amyloid can also appear as serious systemic diseases in which the precursor proteins circulate in plasma and then deposit in a variety of organs. Deposits affect many or most tissues in the body in systemic amyloidosis. Presently, 18 different proteins have been identified as fibril-making in human systemic amyloidoses, each typical of one or a few kinds of disease, hereditary or sporadic^9^. The most common are those with fibrils derived from transthyretin, immunoglobulin light chains (AL), and from serum amyloid A (SAA).

AA amyloidosis is usually a result of a chronic severe, infectious or non-infectious inflammatory disease^10^. The parent protein is serum amyloid A (SAA), in human an acute phase reactant. Two clinically distinct human variants of AA amyloidosis occur, common and vascular, respectively^11–14^. In the common form, glomerular involvement is conspicuous while in the rarer vascular type, amyloid deposits are massive in the renal medulla and in arteries throughout the body, while glomeruli are spared. Patients with the latter pattern develop little or no proteinuria and may, therefore, be diagnosed late in their disease. Congophilia and birefringence is strong in the vascular form but more variable in the common AA amyloidosis. Acute phase SAA is expressed by two different genes, SAA1 and SAA2^15,16^ but the precursor SAA in both vascular and common forms is expressed by the same gene (SAA1) and the fibril proteins differ only by cleavage points of SAA. Presently, there is no explanation to the existence of two distinctly different forms of AA amyloidosis. Cryo electron microscopic studies have shown that the fibrils in the two types are differently folded^17,18^.

In an effort to further uncover a possible strain phenomenon in AA amyloidosis, we have now studied fibrils from the two forms of human AA amyloidosis. We compared the fibril morphology by electron microscopy and the staining properties of amyloid within tissues. With the aid of hyperspectral microscopy of amyloid laden tissues (positive for Congo red) we applied a new method combining the fluorescent Congo red analogue X-34 with a conformation-sensitive luminescent-conjugated oligothiophene, HS-310 to distinguish fibril distributions. We have also used isolated human AA and AL fibrils in in vivo seeding experiments in a murine model of AA amyloidosis^19^ in order to explore whether AA deposits of different properties can be induced.

## Materials and methods

### Human tissue material

Formalin-fixed and paraffin-embedded renal tissue blocks from patients with vascular and common types were available and used for immunohistochemistry (IHC) and immune electron microscopy. Formalin-fixed and paraffin-embedded tissue blocks from other patients were used for the hyperspectral microscope imaging studies (Table 1).

**Table 1:** Human tissues studied by histology and hyperspectral microscope imaging.

Case no	Amyloid subtype	Cause of AA amyloidosis	Tissues in blocks
6	AL kappa	na	Myocardium, pancreas, kidney x 2
7	AL lambda	na	Myocardium, intestine, kidney
8	AL kappa	na	Myocardium, intestine, spleen, bone marrow x 2
9	AL lambda	na	Bone marrow
10	AA vascular	RA	Kidney
11	AA vascular	RA	Kidney
12	AA common	RA	Intestine
13	AA common	RA	Kidney
14	AA vascular	RA	Kidney
15	AA vascular	RA	Lung

*na* not applicable, *RA* rheumatoid arthritis.

Splenic amyloid fibrils from three patients with common AA amyloidosis, one with vascular type AA amyloidosis and one with AL amyloidosis were included in the transmission study (Table 2). All amyloids had been characterized by IHC or western blot analysis and three in addition by amino acid sequence analysis^11,14,20^, including the AL protein which was of immunoglobulin lambda light chain subgroup I origin. Fibrils were extracted as described^21^.

**Table 2:** Results from the transmission experiment.

Case no.	Amyloid subtype	Cause of AA amyloidosis	Animals with amyloid/total	Mean amyloid grade
1	AA common	RA	9/9	2.6 ± 0.5
2	AA vascular	RA	10/10	2.9 ± 0.6
3	AA common	RA	9/9	2.2 ± 1.1
4	AA common	RA	7/9	0.8 ± 0.4
5	AL lambda	na	10/10	2.9 ± 0.6

*na* not applicable, *RA* rheumatoid arthritis.

### Consent statement

The human materials originate from Uppsala Biobank with tissues obtained at autopsy > 30 years ago. Their use for the studies was approved by the Local Ethical Committee at Uppsala University Hospital (01–083). A written consent was not applicable for these materials and identification of individual patients is not possible. All the study methodologies conform to the standards set by the Declaration of Helsinki. The study is reported in accordance with ARRIVE guidelines and all methods were performed in accordance with relevant guidelines and regulations.

### Antibodies and immunohistochemistry

The anti-human SAA monoclonal mouse antibody Sne5 has been characterized earlier^22^. This antibody is directed against an epitope in the N-terminal part of SAA1^23^. Rabbit antiserum 126 was raised against a synthetic peptide MREANYIGSDK, corresponding to SAA1 positions 24–34 while rabbit antiserum 144 was raised against a synthetic peptide DPNHFRPAGLPEKY which corresponds to the C-terminal end of SAA1 (SAA91-104). Peptides were bound to keyhole limpet haemocyanin and rabbit antisera were raised as described^24^. Antiserum against mouse AA has been described earlier^25^. Congo red staining and immunohistochemistry were performed as described earlier^26,27^.

### Electron microscopy

Small pieces of paraffin-embedded material were deparaffinized with xylene, hydrated by decreasing concentrations of ethanol, refixed in 2.5% glutaraldehyde and postfixed in 1% OsO4 and then embedded in Epon. Ultrathin sections were immunolabelled with antisera 126 and 144 and reactivity was visualized with 10 nm gold particles. Samples were examined in a Hitachi electron microscope at 80 kV.

### Transmission experiments

Studies were performed on female NMRI mice, 6–8 weeks old, obtained from B & K Universal, Södertälje, Sweden. The mouse model for AA amyloid was used, where inflammation is induced with subcutaneous injections of silver nitrate, following a previously described protocol^28^. With this protocol mice of the used strain never develop AA amyloidosis within 5 weeks unless given fibril extract (often referred to as amyloid enhancing factor, AEF). Shortly, mice were on day 0 receiving 0.1 ml of diluted suspension (approximately 1 mg/ml) of fibrils from one of the five human amyloid fibril preparations (4 AA and 1 AL), with 10 animals in each group. Inflammation was triggered by a subcutaneous injection of 0.1 ml of 1% silver nitrate on day 0, and repeated on day 7, and 14 and mice were sacrificed on day 16. Isoflurane was used as an inhalation anesthetic before subcutaneous injections.

Animals were killed by cervical dislocation, and 50% of the spleen from each animal was used for squeeze preparation for determination of the degree of amyloid deposition^28^ according to the following grading: 0; no amyloid; 1 + trace of amyloid; 2 + small amyloid deposits; 3 + moderate amyloid deposits; 4 + extensive amounts of amyloid. The rest of spleen and both kidneys were fixed in formalin, embedded in paraffin, sectioned and stained with Congo red for studies of amyloid deposition distribution. Sections were also immunostained with antisera A126, A144 or antiserum against mouse AA. All animal experiments were approved by the local Animal Ethics Committee at Uppsala University and conducted according to the regulations of Uppsala University, Sweden.

### Comparison of protein AA species seeded in the mouse by human AA and AL amyloid

Section (10 μm thick) from formalin-fixed paraffin-embedded spleen placed in Eppendorf tubes were deparaffinized with xylene, rinsed with absolute ethanol, and dried. The tissue was incubated with 200 µl of formic acid for 6 h, and after centrifugation, 100 µl of the clear supernatant was recovered and dried under nitrogen. The protein film formed was solubilized in 50 µl dimethyl sulfoxide, followed by adding 100 µl SDS-PAGE sample buffer (100 mM Tris·HCl, pH 6.8, 4% SDS, 12% (v/v) glycerol, 4 mM dithiothreitol, 0.05% (w/v) Bromophenol blue) and incubating at 95^o^C for 5 min. The samples were separated on 12% PAGE and transferred to a nitrocellulose paper (NP) using an Iblot 2 device. Antigenic retrieval was performed by placing the NP in boiling 50 mM Tris HCl buffer pH 7.4 with 150 mM NaCl (TBS) for 5 min, and unoccupied binding sites on the paper were blocked by incubating in 5% milk in TBS-0.1% Tween-20 (TBS-T) for 60 min. Murine protein AA was detected with a primary antibody raised in rabbits diluted 1:500 in TBS-T^25^, and detection antibody HRP labeled goat anti-rabbit diluted 1:1500. Immobilon chemiluminescent HRP substrate (Merck) was used for visualization.

### Fluorescent amyloid ligands

The fluorescent ligand X-34, a Congo red analogue (2,5-bis(4’-hydroxy-3’-carboxy-styryl)benzene)^29,30^) and HS-310^31^ a luminescent conjugated oligothiophene with six thiophene rings were used for co-staining of amyloid tissues. These molecules do not have specificity for a certain protein but bind to surface grooves within β-sheet fibrils. The binding produces unique fluorescent patterns and can separate differences in fibril structure^32^. Deparaffinized sections of amyloid laden human tissues and mouse spleens were incubated for 30 min with a mixture of X-34 and HS-310 (final concentration 0.5 µM of each) in 0.01 M phosphate buffer, pH 7.4 with 0.14 M NaCl (PBS) and then washed in PBS. Hyperspectral microscope imaging using a Leica 6000B fluorescence microscope equipped with a spectral camera (Applied Spectral Imaging, Migdal-Ha-Emek, Israel) was used to analyze perifollicular amyloid in the mouse splenic sections and human tissue sections with 405 nm excitation filter and a long pass emission filter. From the hyper spectral images, the fluorescence emission ratio between X-34 (measured at 471 nm) and HS-310 (measured at 552 nm) values were obtained for regions of interest within the tissue images. Values from the different groups were compared.

### Statistics

Fluorescence emission ratio comparisons between the groups of human systemic amyloidosis tissues and spleen follicular amyloid from seeded AA mice respectively were done with ordinary one-way Analysis of variance (ANOVA) with Tukey’s multiple comparison test between all groups. Significance was set at *p* < 0.05. All calculations were performed in GraphPad Prism 10. Statistical data and analyses are shown in supporting information.

## Results

### Amyloid deposits as visualized with the two antibodies Sne5 and A144

**Fig. 1:**
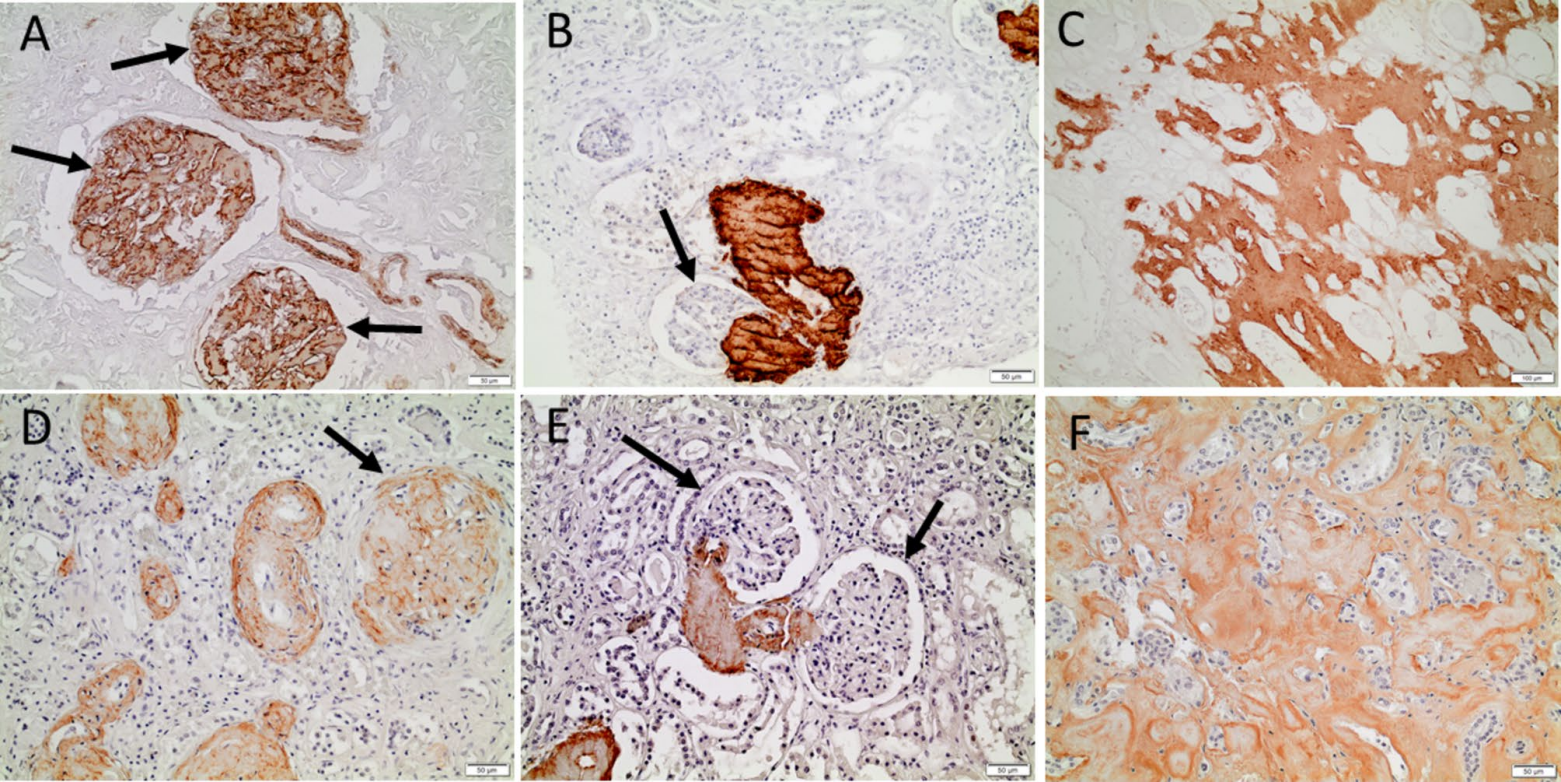
Human AA amyloid deposits visualized by immunohistochemical staining, in (**A-C**) with mouse monoclonal antibody Sne5, directed against an N-terminal epitope and in (**D-F**) with antiserum A144 against the C-terminus of SAA. Renal amyloid of the common type is seen in (**A**) and (**D**) while the vascular type is shown in (**B**,** C**,** E** and **F**). Note absence of glomerular amyloid in (**B**) and (**E**) despite large amount of amyloid in arterioles. Bar in (**A**, **B**, **D**, **E** and **F**) 50 μm and in (**C**) 100 μm.

The mab Sne5 labelled all AA amyloid of vascular and common types in human tissue strongly and evenly (Fig. 1, A-C). Antiserum A144, reactive against the C-terminal region of SAA1 gave a weaker reaction and a more uneven labelling but with a stronger reactivity at the external parts of deposits in all studied organs (Fig. 1, D-F) and there were no differences between the common or vascular types.

### Fibril morphology in situ of the two AA amyloid forms

**Fig. 2:**
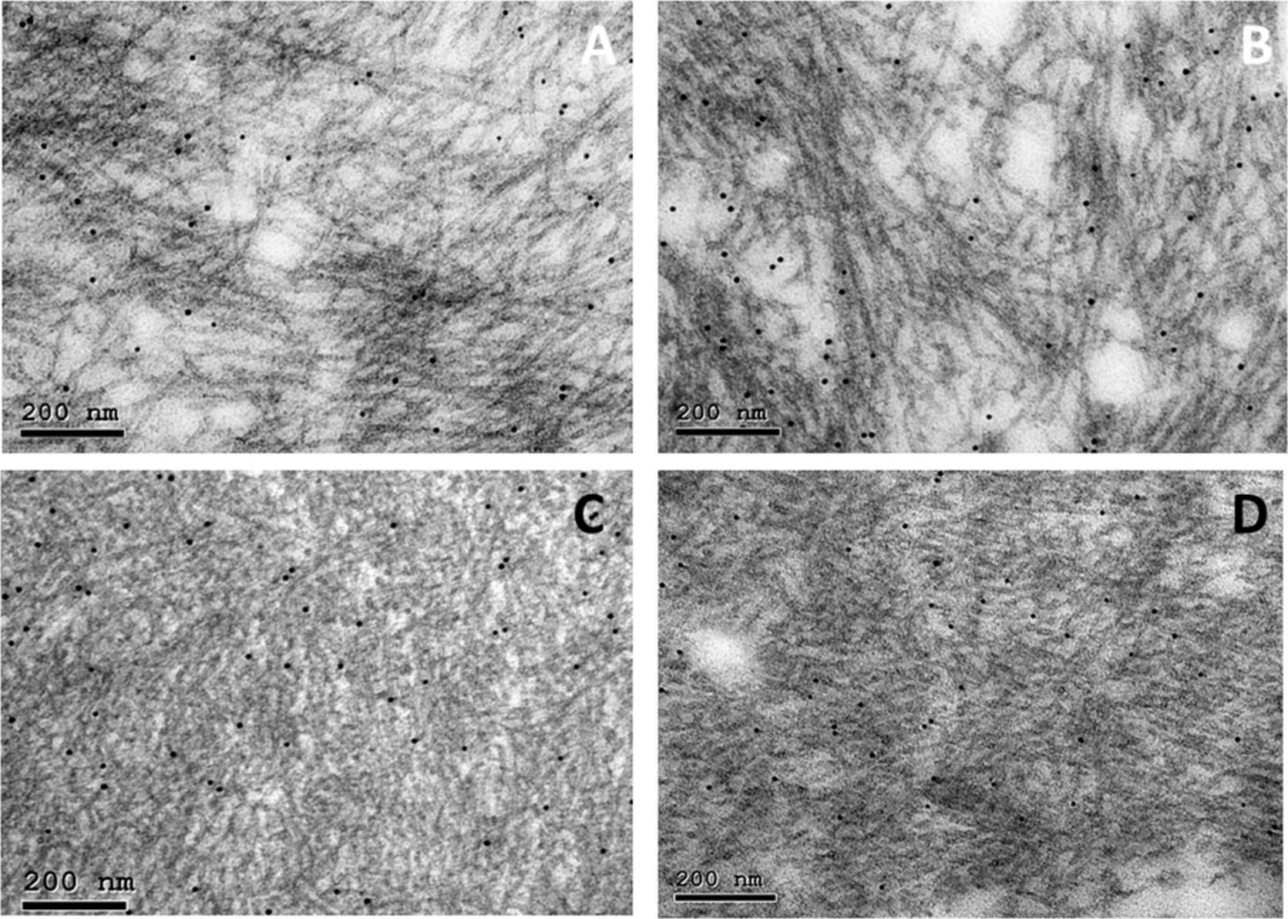
Electron microscopical appearance of AA amyloid of vascular type (**A** and **B**) and of common type (**C** and **D**). Fibrils from the vascular type are very distinct and well separated while fibrils are more tightly packed in (**C** and **D**). Immunolabelled with antiserum A126 against protein AA, 10 nm gold particles. Bar 200 nm.

Representative electron microscopic pictures of the studied four materials (two of vascular and two of common type of AA amyloid) are shown in Fig. 2. Fibril dimensions showed no definite difference between the two types, but the exact measurement is difficult on sectioned material. However, fibrils from the vascular type of deposition (Fig. 2A and B) were more distinct and amyloid deposits appeared more clean, without many other components. Vascular type protein AA is a mixture of two distinct components, one being almost full-length SAA1 and the other consisting of the N-terminal 48 amino acid residues, both lacking the N-terminal arginine residue^14^. As seen in Fig. 2A and B, a dominating feature was even, comparably thick (around 10 nm) and very distinct fibrils. However, a careful look will disclose more weakly contrasted and thinner fibrils as well. It is not possible to determine whether the two components were separate fibrils from the two AA variants since in EM, immunolabelling with the anti C-terminal SAA antiserum A144 was too weak. Fibrils in deposits from the common form of AA amyloidosis (Fig. 2C and D) tended to be less distinctive, to stick together and mixed with other tissue components, including collagen fibers. A feature that distinguished fibrils of vascular from common type was the length of individual fibrils: very long fibrils were evident in vascular deposits.

### Spectral analyses of human amyloid

**Fig. 3:**
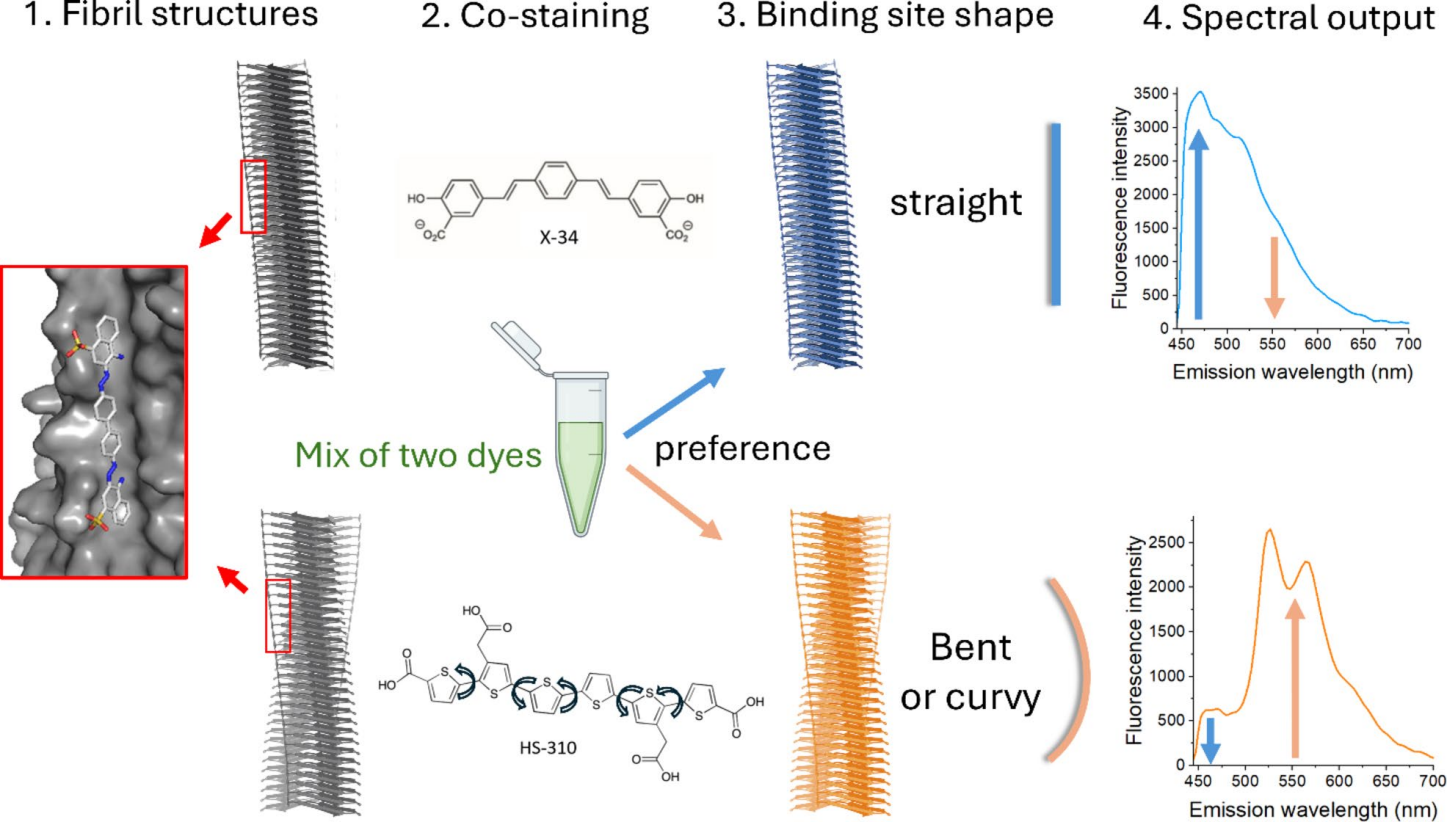
Schematic figure of the hypothesis of distinguishing amyloid fibril structures by amyloid dyes. (1) Two different amyloid fibril structures both contain the Congo red amyloid binding site (illustrated by PyMol of PDB 2LBU from^33^). (2) Co-staining with the rigid X-34 (blue fluorescent) and the flexible (HS-310) amyloid dye demonstrated by free rotations between the thiophene rings. Both ligands preferably binds to the Congo red binding site on the fibril. (3) Shape of the binding site determines preference. (4) The readout of the analysis is performed by the spectral output of the stained tissue amyloid deposits with hyperspectral microscopy by the comparative intensity of the 471 nm emission peak for X-34 (blue) and 552 nm peak for HS-310 (orange). The arrows indicate the spectral output for the respective fibril binding site preference.

**Fig. 4:**
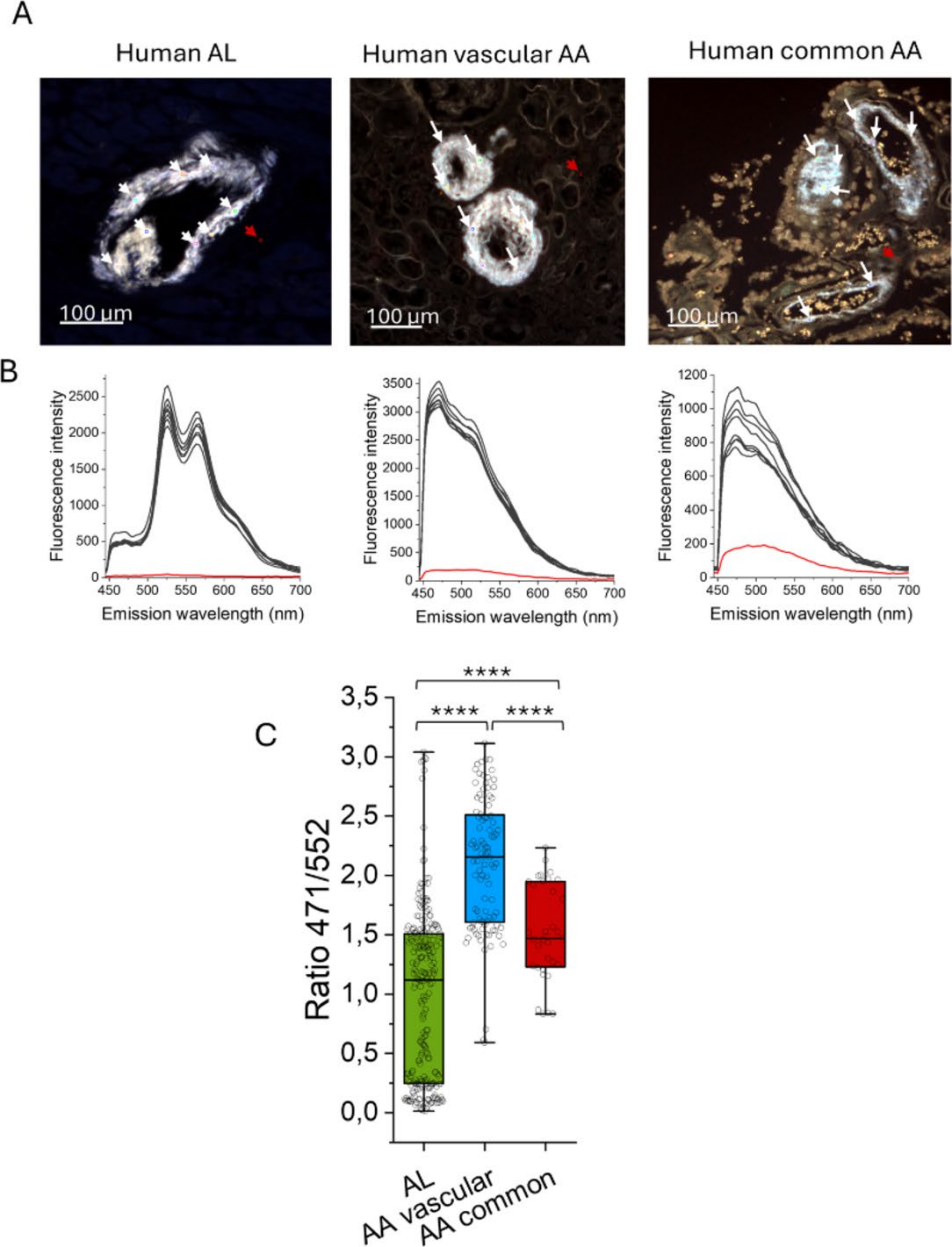
Fluorescent amyloid ligand combinations for discrimination of amyloid structures in human tissue. (**A**) Hyper spectral fluorescence micrographs of human tissue with systemic amyloidosis co-stained with X-34 and HS-310. Amyloid is clearly visible. Regions of interest (ROI) were selected as indicated by arrows and emission spectra from each ROI are displayed in (**B**). White arrows in (**A**) depict spectra (black lines) within the amyloid, red is background of surrounding tissue. X-34 emits with a peak centered at 460–480 nm and HS-310 with a double peak in the interval 530–570 nm. (**C**) Fluorescence ratio of X-34 and HS-310 emission at 471/552 nm for ROIs from the different groups of tissues. AL = 13 tissues from 4 patients. AA vascular = 2 tissues from 2 patients. AA glomerular (common) = 6 tissues from 4 patients. Statistics in C: One-way ANOVA with Tukey’s test comparing all groups: *p < 0.05; **p < 0.01; ***p < 0.001; and ****p < 0.0001. *ns* nonsignificant.

We analyzed amyloid in several human tissues using a combination of the amyloid dyes X-34 and HS-310. Both dyes bind to the Congo red binding site outlined as a groove along the fibril axis on the surface of amyloid fibrils^33–35^ (Fig. 3). The fluorescent amyloid ligands fluoresce with distinct emission spectra allowing a discrimination of preference of ligand binding. The difference between the ligands is that X-34 is a rigid straight trans-stilbene scaffold, whereas HS-310 is a flexible molecule with free-rotation between its six thiophene units (Fig. 3). Our hypothesis was that a straight binding pocket will stain preferentially with the rigid X-34 dye while a more bent, curved, or humped binding pocket will preferentially stain with HS-310 which can more easily accommodate such molecular variations (Fig. 3). Amyloid structure should thereby be distinguishable by hyperspectral microscopy of the amyloid in tissue stained with equimolar concentrations of both dyes and imaged with the same excitation wavelength. The staining patterns of AL and AA vascular and AA common human tissue sections were assessed (Table 1; Fig. 4A,B). The pattern of staining was diverse between patients and between tissues rendering a spread of fluorescence signal of X-34 and HS-310 analyzed by ratiometric intensity comparisons. The largest variation was seen within AL amyloidosis (Fig. 4C). Statistical analysis (ANOVA and Tukey’s test) revealed significant differences between these three amyloidosis groups (Fig. 4C). AL amyloid displayed a significantly lower mean ratio of X-34/HS-310 fluorescence while the mean for AA was higher (Fig. 4C). The differences were also significant between AA vascular and AA common.

### Transmission experiments

Extracted fibril material from 5 patients with systemic amyloidosis accelerated splenic amyloid formation in mice (Table 2) without any noticeable difference in efficacy, with the exception of fibrils from one common AA case (Case no. 4). Recipient mice obtaining this material had a significantly less amount of amyloid compared to all other groups and in this group two mice appeared which were completely devoid of any deposits. There is no clear explanation for this finding. In all other animals, amyloid outlined lymph follicles and colocalized with silver grains. There were no evident morphological or Congo red staining differences between the animal groups. No prominent vascular deposits were seen in any animal.

**Fig. 5:**
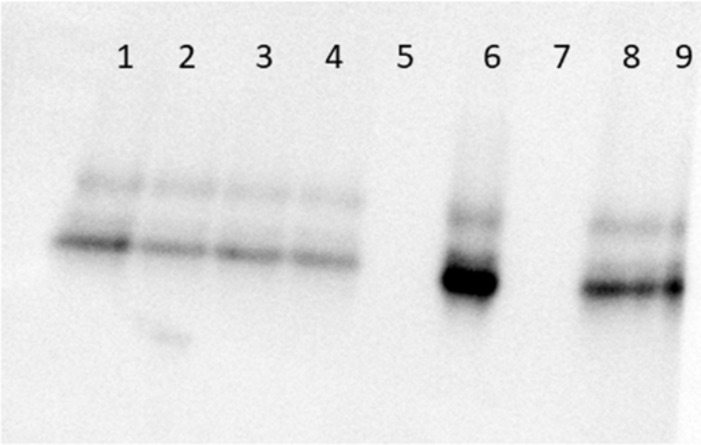
Protein AA has identical size in all recipient mice. SDS-PAGE of extracts from murine splenic sections followed by western blot with an anti-mouse AA antiserum. 1–3 show protein AA induced with AL fibrils, 4–6 with common AA amyloid fibrils and 7–9 with vascular AA amyloid fibrils. The amount of amyloid varied and there was no amyloid left in materials from mouse No. 5 and 7.

From the western blot, it is evident that seeding AA amyloid with the five AEF extracts (three common AA, one vascular AA and one AL amyloid) did not result in differences in protein size distribution. Instead, the reactivity patterns are identical (Fig. 5). The samples were separated on the same SDS-PAGE, but since the signal intensity varied, the increment mode was used for capturing the images, and the presented result is a combination of two different images. Earlier MS analysis of murine protein AA revealed a predominating fragment corresponding to 82 residues (9.3 kDa)^19^.

**Fig. 6:**
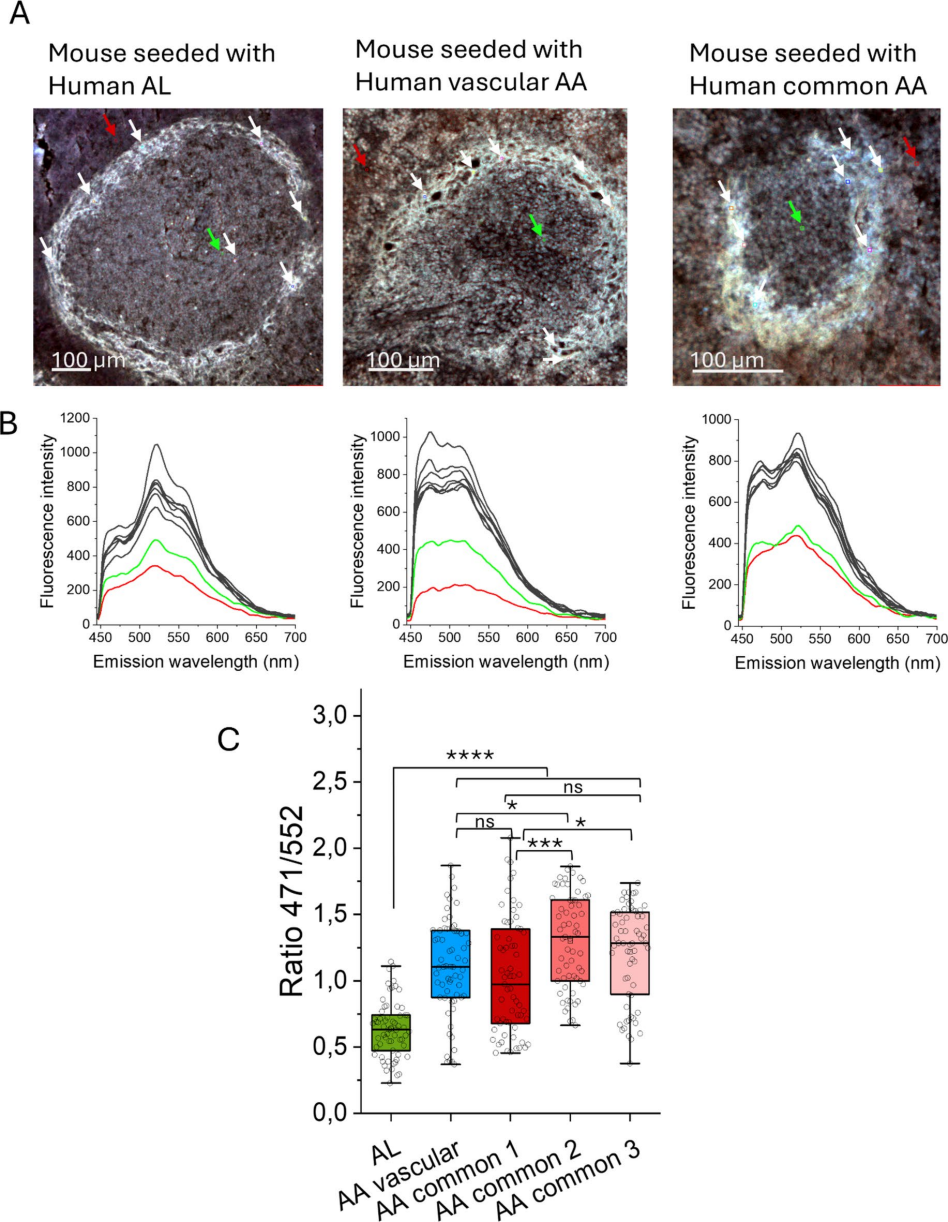
Fluorescent amyloid ligand combinations for discrimination of amyloid structures in mouse tissue. (**A**) Hyper spectral fluorescence micrographs of AA mouse spleen co-stained with X-34 and HS-310. Amyloid is clearly visible. Regions of interest (ROI 5 × 5 pixels) were selected as indicated by arrows and emission spectra from each ROI are displayed in (**B**). White arrows are depict spectra (black lines) within the amyloid follicle rim, red is outside of follicle and green is inside. X-34 emits with a peak centered at 460–480 nm and HS-310 with a double peak in the interval 530–570 nm. (**C**) The fluorescence ratio of X-34 and HS-310 emission at 471/552 nm for ROIs from the different groups of mice (*n* = 3 in each group). Statistics in C: One-way ANOVA with Tukey’s test comparing all groups: *p < 0.05; **p < 0.01; ***p < 0.001; and ****p < 0.0001. *ns* nonsignificant.

We next analyzed the amyloid around splenic lymph follicles of three mice from each mouse group using the combination of the amyloid dyes X-34 and HS-310 (Fig. 6A) as described previously for human samples. The amyloid fluorescent ligands fluoresce with distinct emission spectra allowing a discrimination of preference of ligand binding (Fig. 6B). Statistical analysis (ANOVA and Tukey’s test) of the intensity ratios between X-34 and HS-310 showed a significant difference (*p* < 0.0001) between the group of mice, seeded with AL fibrils and each of the four AA seeded groups (Fig. 6C). When comparing the group of mice seeded with fibrils from deposits of vascular type AA against the three groups given material from common AA amyloid there was a significant difference (*p* = 0.018) only for one group (mice seeded with fibrils from case 4, Table 2). There were also statistical significant differences between groups of mice seeded with separate AA common types, so no clear pattern was resolved (Fig. 6C). The comparative staining pattern between mice seeded with AL compared to mice seeded with either type of AA fibrils mirrors the pattern observed in the human material (*c.f*. Figures 4C and 6C). This correlation supports that seeding has to some extent templated formation of amyloid structure in the recipient mice. Notably the human tissues in the study were not the same as the donor material used for seeding except for one case of AA amyloidosis.

## Discussion

‘One of the central principles in biology and biochemistry is that protein structures are fully and uniquely determined by amino acid sequences. This principle does not apply to amyloid fibrils’^36^. Although a beta-sheet structure with hydrogen bonding between the peptide backbones are characteristic of all amyloid fibrils, there are many possibilities of variations such as parallel or anti-parallel organization of β-strands, length and organization of non-β-strand domains, side-chain interactions and so on^37^. Most interestingly, these variations in fibril structures are self-propagating by seeding^3^. However, the molecular mechanism behind it is poorly understood. Seeding is generally very specific but induction of AA amyloidosis in inflamed mice can be enhanced by different amyloid fibrils, both derived from nature and produced artificially in the laboratory^28,38,39^ but not as efficiently as with murine AA fibrils^19^. Exactly how non-identical fibrils exert this cross-seeding in amyloidogenesis in recipients is unclear^37^ and one possibility is ‘surface assisted nucleation^40^, which is believed to depend on interactions between particles, in our case fibrils, with monomers of similar or different proteins. With such a nucleation mechanism, an amyloid accelerating effect of AL fibrils on AA amyloidogenesis should not be surprising.

Although human AA amyloidosis is regarded as a relatively uniform disease with symptoms particularly from the kidneys, there are human inter-individual variations both in symptoms and in distribution patterns in different organs^10^. In addition to the variations in renal tissues there are rare examples of large deposits in the thyroid or the heart. The reasons behind these phenotypes are mainly unknown.

The vascular and common forms of AA amyloid differ in their light microscopic appearance^10^ and we show now that also their ordinary ultrastructural appearance is clearly different although the fibril protein comes from the same gene product and they differ in length of the peptides, depending on differences in cleavage points^10,13^. We have suggested that the two variants of human AA deposits may be example of strains in a systemic amyloidosis form^10,41^.

With the aid of a flexible conformation-sensitive luminescent-conjugated oligothiophene and the rigid Congo red analogue X-34 we analyzed the two forms of AA amyloidosis as well as one case of AL amyloidosis. There were significant differences between the fluorescence signals of the AL material and the AA deposits and there was also a significant difference between the vascular and common AA types. The vascular AA amyloid fibrils rendered a higher X-34 binding than common AA fibrils suggesting higher presence of straight and rigid fibrils in the vascular AA amyloid. The fluorescence data hence correlated with the observations from electron microscopy, implicating differences in structure within the two types of human AA fibrils.

We have previously shown that two different murine AA fibril isolates (AEF) displayed a significant difference in amyloid ligand fluorescence by staining with two luminescent conjugated oligothiophenes. Mice with AA amyloidosis induced by the two AA fibril seeds displayed similar features during Congo red analysis and grading of amyloid load. Nonetheless, the analyzed tissue samples displayed highly significant differences in spectral signatures when stained with a combination of amyloid dyes which indicated differences in deposited AA conformation, mirrored by the seed spectral profiles, i.e., the presence of different amyloid strains in the AA fibril seed isolates^42^.

We did not obtain clear different mouse AA amyloid fibril strains by seeding with human AA fibrils of different types with the analyzing techniques we used here. One may argue that there are pronounced differences in molecular organization in human and mouse AA fibrils^17^ but the N-terminal segment consisting of about positions 1–21, important in fibrillogenesis^43^ are similarly folded in both species. It may be the case that induced templating during seeding does not occur in mouse AA. However, surprisingly and in contrast with seeding with the two human AA fibril types, induction of AA amyloidosis in mice with human AL fibrils generated splenic AA deposits of another type, as compared with the other groups of mice induced with human AA fibrils. All three mice in the AL-fibril-receiving group gave similar results rendering fibril types which preferred HS-310 staining, suggesting a less organized fibril structure compared to that formed after human AA seeding. This finding supports the hypothesis that there may exist strains also in AA amyloidosis but that the differences in molecular organizations can be subtle. Since many different amyloid ligands with variations in binding properties can be synthesized^44^ it may be possible to develop molecules and techniques to differentiate between fibril variants.

### Limitations

There are some limitations in our study. First, it had been interesting to study AA fibrils derived from more patients and isolated from different organs. Secondly, it had also been of great interest to use AL fibrils from patients with different genotypes. It would also be preferred that the amyloid seed donor material was stained by the same protocol as was used for the inoculated mice.

## Conclusions

Fibrils in two types of human AA amyloidosis are differently organized, evident already in conventional electron microscopy. Transmission experiments in a mouse model indicate that AA fibril strains exist and can be efficiently propagated. Cross-seeding with non-homologous fibrils accentuated fibril differences formed in recipient mice. This finding is important and may partially explain the existence of different phenotypes in human AA amyloidosis.

## Electronic supplementary material

Below is the link to the electronic supplementary material.

Supplementary Material 1

## Data Availability

The datasets analyzed during the current study are available from the corresponding author on reasonable request.

## Abbreviations

AA: Amyloid A
AEF: Amyloid enhancing factor
AL: Amyloid light chain
ATTR: Amyloid transthyretin
NMRI: Naval Medical Research Institute
RA: Rheumatoid arthritis
SAA: Serum amyloid A
