# Supplementary material for: The question of strains in AA amyloidosis
Source: Sci Rep. 2025 Jan 29;15:3684. doi: 10.1038/s41598-025-87239-6 (PMC11779915; doi:10.1038/s41598-025-87239-6)
Supplement: Supplementary file 1 — Supplementary Material 1 [file 41598_2025_87239_MOESM1_ESM.docx]

**Statistical analysis of X34/HS310 amyloid fluorescence spectra 417/552 nm**

Method: Ordinary one-way ANOVA with Tukey’s multiple comparison test between all groups

**Human amyloidosis**

| **Tukey's multiple comparisons test (human amyloidosis)** | **Mean Diff.** | **95 % CI of diff.** | **Below threshold?** | **Summary** | **Adjusted P Value** |
| --- | --- | --- | --- | --- | --- |
| AL vs. AA vascular | -0,5482 | -0,8409 to -0,2555 | Yes | **** | <0,0001 |
| AL vs. AA common | -1,115 | -1,303 to -0,9272 | Yes | **** | <0,0001 |
| AA vascular vs. AA common | -0,5668 | -0,8843 to -0,2493 | Yes | **** | <0,0001 |

**Seeded mouse amyloidosis**

| **Tukey's multiple comparisons test (seed groups)** | **Mean Diff.** | **95 % CI of diff.** | **Below threshold?** | **Summary** | **Adjusted P Value** |
| --- | --- | --- | --- | --- | --- |
| AL vs. AA vascular | -0,4695 | -0,6420 to -0,2971 | Yes | **** | <0,0001 |
| AL vs. AA common 1 | -0,3992 | -0,5716 to -0,2267 | Yes | **** | <0,0001 |
| AL vs. AA common 2 | -0,6638 | -0,8363 to -0,4914 | Yes | **** | <0,0001 |
| AL vs. AA common 3 | -0,5809 | -0,7533 to -0,4084 | Yes | **** | <0,0001 |
| AA vascular vs. AA common 1 | 0,07036 | -0,1021 to 0,2428 | No | ns | 0,7962 |
| AA vascular vs. AA common 2 | -0,1943 | -0,3667 to -0,02182 | Yes | * | 0,0184 |
| AA vascular vs. AA common 3 | -0,1113 | -0,2838 to 0,06112 | No | ns | 0,3922 |
| AA common 1 vs. AA common 2 | -0,2646 | -0,4371 to -0,09218 | Yes | *** | 0,0003 |
| AA common 1 vs. AA common 3 | -0,1817 | -0,3541 to -0,009241 | Yes | * | 0,0333 |
| AA common 2 vs. AA common 3 | 0,08294 | -0,08951 to 0,2554 | No | ns | 0,6791 |
